# Novel value-added uses for sweet potato juice and flour in polyphenol- and protein-enriched functional food ingredients

**DOI:** 10.1002/fsn3.234

**Published:** 2015-04-09

**Authors:** Mary H Grace, An N Truong, Van-Den Truong, Ilya Raskin, Mary Ann Lila

**Affiliations:** 1 Department of Food, Bioprocessing and Nutrition Sciences, Plants for Human Health Institute, North Carolina State University, North Carolina Research CampusKannapolis, North Carolina, 28081; 2 Department of Food, Bioprocessing and Nutrition Sciences, North Carolina State UniversityRaleigh, North Carolina, 27695; 3 USDA-ARS, SAA Food Science Research Unit, Department of Food, Bioprocessing and Nutrition Sciences, North Carolina State UniversityRaleigh, North Carolina, 27695; 4 Department of Plant Biology & Pathology, SEBS, Rutgers UniversityNew Brunswick, New Jersey, 08901

**Keywords:** Anti-inflammatory, antioxidant, berries, polyphenols, stability, sweet potato

## Abstract

Blackcurrant, blueberry, and muscadine grape juices were efficiently sorbed, concentrated, and stabilized into dry granular ingredient matrices which combined anti-inflammatory and antioxidant fruit polyphenols with sweet potato functional constituents (carotenoids, vitamins, polyphenols, fibers). Total phenolics were highest in blackcurrant-orange sweet potato ingredient matrices (34.03 mg/g), and lowest in muscadine grape-yellow sweet potato matrices (10.56 mg/g). Similarly, anthocyanins were most concentrated in blackcurrant-fortified orange and yellow sweet potato matrices (5.40 and 6.54 mg/g, respectively). Alternatively, other protein-rich edible matrices (defatted soy flour, light roasted peanut flour, and rice protein concentrate) efficiently captured polyphenols (6.09–9.46 mg/g) and anthocyanins (0.77–1.27 mg/g) from purple-fleshed sweet potato juice, with comparable efficiency. Antioxidant activity correlated well with total phenolic content. All formulated ingredient matrices stabilized and preserved polyphenols for up to 24 weeks, even when stored at 37°C. Complexation with juice-derived polyphenols did not significantly alter protein or carbohydrate profiles of the matrices. Sensory evaluation of the ingredient matrices suggested potential uses for a wide range of functional food products.

## Introduction

Worldwide, sweet potato (*Ipomoea batatas* L.) is the sixth most important food crop and is a staple food source for many indigenous populations in Central and South America, Japan, Africa, the Caribbean, Polynesia, Hawaii, and Papua New Guinea. North Carolina is the main producer of sweet potato in the USA and accounts for more than 40% of the national supply (NCDA & CS 2012). The sweet potato is an excellent source of natural health-promoting compounds for the functional food market, such as phenolic acids, vitamins, *β*-carotene, and anthocyanins (Grace et al. 2014b). Also, the high concentration of highly stable acylated anthocyanins suggests that purple-fleshed sweet potato may be a promising natural alternative to synthetic coloring in food products (Truong et al. 2010).

Although plant foods and vegetables do not contain vitamin A as such, they can contain precursors, such as pro-vitamin A (*β*-carotene and other carotenoids) that the human body can convert to vitamin A. Orange-fleshed sweet potato (such as Covington genotype) is an excellent source of *β*-carotene (Grace et al. 2014b), responsible for alleviating vitamin A deficiencies and night blindness (van Jaarsveld et al. 2005). In addition, *β*-carotene has demonstrated antioxidant and anticancer activities and may help in protection against coronary heart diseases (Mayne 1996). Yellow- and orange-fleshed sweet potato contain a blend of phenolic acids including hydroxycinnamic acids, but they are deficient in the major polyphenolic phytochemicals present in berry fruits, for example, anthocyanins, proanthocyanidins, and ellagitannins. Purple-fleshed sweet potato, however, has high levels of acylated anthocyanins and other phenolics with antioxidant and anti-inflammatory activities (Grace et al. 2014b). These anthocyanins are not only highly stable but also provide health-related radical-scavenging activity (Oki et al. 2002), memory-enhancing effects (Cho et al. 2003; Shan et al. 2009; Lu et al. 2012), and hepatoprotective activity (Zhang et al. 2009; 2013).

Recently, we introduced a novel strategy that enabled rapid and streamlined one-step separation of polyphenolic compounds in fruit and vegetable juices from extraneous or high caloric components and water (Roopchand et al. 2012a; Grace et al. 2013), via sorption to protein-rich substrates (e.g., soy, hemp, or peanut flours) to produce stable dry powdered ingredient matrices. Formulated lightweight, stable dry functional concentrated phytoactive- and protein-enriched matrices were particularly well suited for portable functional foods applications including combat rations, camping, or lunch boxes (Grace et al. 2014a; Yousef et al. 2014). Fruit polyphenols were stabilized and protected by complexation to protein-rich food matrices during transit through a simulated digestive tract (Ribnicky et al. 2014) and in vivo, where they significantly lowered blood glucose in obese and hyperglycemic mice (Roopchand et al. 2013). Clinical administration of the polyphenol-enriched protein matrices to athletes resulted in enhanced ketogenesis during recovery from 3 days heavy exertion (Nieman et al. 2013), and significant protection from exercise-induced susceptibility to virus infections (Ahmed et al. 2014). The same technology was also adopted as a strategy to combat malnutrition in rural village communities in Zambia, by stabilizing perishable, seasonal fruits into protein flour matrices (Guzman et al. 2014).

In this study, we adapted the approach to develop novel value-added uses for sweet potato through (1) fortification of orange and yellow sweet potato flours with concentrated anthocyanins and other polyphenols from fruits; and (2) capturing and complexing polyphenols from purple-fleshed sweet potato water extracts to other protein-rich edible matrices. The polyphenol-fortified sweet potato food ingredient matrices were evaluated in terms of antioxidant capacities (using three different assays), nutritional proximate analysis, sensory properties, and polyphenol stability over time.

## Materials and Methods

### Chemicals

Cyanidin-3-*O*-glucoside was purchased from Chromadex (Irvine, CA). 2,2-Diphenyl-1-picrylhyrazyl (DPPH), Trolox (6-hydroxy-2,5,7,8-tetramethylchromane-2-carboxylic acid), 2,2′-azino-bis(3-ethylbenzothiazoline-6-sulfonic acid) diammonium salt (ABTS), 2,4,6-tripyridyl-s-triazine (TPTZ), FeCl_3_.6H_2_O, gallic acid, and Folin–Ciocalteu's phenol reagent were purchased from Sigma-Aldrich Inc. (St. Louis, MO). All organic solvents were HPLC grade and obtained from VWR International (Suwanee, GA).

### Protein-rich matrices

Orange-fleshed sweet potato (cv. Covington) and a yellow-fleshed mutant referred to as Yellow Covington were grown at the experimental fields of the Sweet Potato Breeding Program (Clinton, NC), North Carolina State University. The harvested roots were cured at 30°C, 85–90% relative humidity for 7 days, and stored at 13–16°C, 80–90% relative humidity for 4 months prior to sampling for the experiments. The roots (40 kg) were peeled and cut into 0.5-cm-thick slices. Blanching was carried out using steam at 100°C for 3 min, and then the slices were spread on mesh trays for drying in a cabinet dryer at 65°C for 24 h (Truong and Avula 2010). The dried chips were ground into fine flour using a flour mill (Model 91- Kitchen Mill; Blendtec Company, Orem, UT). The orange and yellow sweet potato flour samples (OSP and YSP, respectively) were bagged in plastic bags and stored at −80°C. Other sources of edible proteins included defatted soy flour (DSF, 50% protein) (Hodgson Mill Inc., Effingham, IL), partially defatted peanut flour (12% fat), light roast peanut flour (LPF, Virginia type, 50% protein) (Golden Peanut Company, Alpharetta, GA), and rice protein concentrate (RPC, 70% protein) (Metagenics, Gig Harbor, WA).

### Polyphenol sources

The fruit juices used included: blackcurrant juice concentrate, 65 ºBrix (BC, *Ribes nigrum*) (The New Zealand Blackcurrant Co-operative Ltd, Nelson, New Zealand), blueberry juice concentrate, 50 ºBrix (BB, *Vaccinium angustifolium* Aiton) (Oxford Frozen Foods, Oxford, Canada), and muscadine grape juice concentrate from Noble variety, 50 ºBrix (MG, *Vitis rotundifolia*) (Muscadine Products Corporation, Wray, GA). Purple-fleshed sweet potato juice (PSP) was prepared as follows: the washed tuberous roots of purple-fleshed sweet potato (cv. Stokes Purple) (grown in Bailey, NC) were cut into 2.5-cm segments. The segments (2 kg) were blended with 2 L of 3% citric acid solution for 2 min using a heavy-duty blender (model LBC 15; Waring Commercial, Torrington, CT). The slurry was passed through a juice extractor (Super Angel model 5500; U.S. Juicers, Tustin, CA). The extracted juice was placed in a 4°C cold room overnight to allow raw starch to settle and then the juice was decanted and centrifuged at 10,000 rpm for 15 min at 4°C. The clear undiluted juice (6.4 ºBrix) was dispensed into 200-mL aliquots and stored at −80°C until use.

### Fortification of protein-rich matrices with polyphenols

Fruit juice concentrates (blackcurrant, blueberry, or muscadine grape) were diluted with water (1:3 v/v) before measurement of dry matter, total phenolic, and anthocyanin contents. The methods for sorption of fruit juices to edible protein-rich flours or protein isolates have been previously reported (Grace et al. 2013). Briefly, the sweet potato flour was added to the diluted juice at a concentration of 100 g/L (4 replicates). Each mixture was stirred for 15 min, centrifuged at 14,489 × *g* and the solid pelleted material was freeze-dried, powdered, and stored at −20°C. In parallel trials, the other protein-rich matrices (defatted soy flour, light roast peanut flour, or rice protein concentrate) were added to the purple sweet potato juice extract at a concentration of 100 g/L (4 replicates), and the mixtures were treated as above. Parallel experiments were run using flour matrices with water instead of fruit juices, in order to measure the polyphenols contributed by the flours alone. The dried sweet potato matrices fortified with fruit polyphenols or water, as well as the other protein-rich matrices fortified with purple sweet potato polyphenols, were analyzed for polyphenolic contents, antioxidant capacities, nutritional proximate contents, descriptive sensory properties, and 24-week shelf-stability, as described below.

Three aliquots (0.5 g) from each polyphenol-fortified matrix or water-treated matrix were extracted with 8 mL of 1% acetic acid in 80% methanol in water with sonication for 5 min at 55°C, and then centrifuged for 10 min prior to collection of the supernatant in a 25-mL volumetric flask. The process was repeated on the pellet two more times; the supernatants were combined and brought to 25 mL with the extraction solvent. This extract was used to determine total phenolics, anthocyanins, and antioxidant capacity.

### Measurement of dry matter, total phenolic, and anthocyanin contents

The juices and extracts from the polyphenol-fortified matrices were diluted to appropriate concentrations for analysis. Total solids (dry matter content, DM) were measured in juices by drying a sample in a convection oven at 105°C until constant weight. Total phenolics (TP) were determined with Folin–Ciocalteu reagent by the method of Singleton et al. (1999). Concentrations were expressed as mg/g gallic acid equivalents based on a created gallic acid standard curve. Total monomeric anthocyanin (ANC) was measured by the pH differential spectrophotometric method (Lee et al. 2005), using a Shimadzu UV-2450 (Shimadzu, Kyoto, Japan) spectrophotometer. Anthocyanin concentration was calculated as milligrams per gram cyanidin-3-*O*-glucoside equivalents.

### Free radical-scavenging activity (DPPH assay)

Free radical-scavenging activity was measured using the stable DPPH radical and Trolox as reference substances (Truong et al. 2007). Samples were diluted 10× with 80% methanol before mixing with the reagent. An aliquot (100 *μ*L) of each sample was pipetted into 3.9 mL of DPPH solution (0.08 mol/L in 95% ethanol) to initiate the reaction. After a reaction time of 3 h at ambient temperature the reaction had reached completion. The decrease in absorbance of DPPH free radicals was read at 515 nm against ethanol as a blank using a Shimadzu UV-2450 spectrophotometer. Trolox (0, 100, 200, 300, 400, and 500 *μ*mol/L) was used as a standard antioxidant compound. Analysis was performed in triplicate for each sample and each concentration of standard. The antioxidant activity is reported in *μ*mol of Trolox equivalents per gram matrix (*μ*mol TE/g).

### Free radical-scavenging activity (ABTS assay)

The ABTS assay was performed as described by Re et al. (1999) based on the reduction in ABTS^+•^ radicals by antioxidants in the extracts from the matrices. ABTS radical cation (ABTS^+•^) was produced by reacting ABTS solution (7 mmol/L) with 2.45 mmol/L potassium persulfate (final concentration) and allowing the mixture to stand in the dark at room temperature for 12–16 h before use. For the assay, the ABTS^+•^ solution was diluted in deionized water or ethanol to an absorbance of 0.7 (±0.02) at 734 nm. After the addition of 100 *μ*L of the matrix extract to 3 mL of ABTS^+•^ solution, the absorbance reading was taken at 30°C, 10 min after initial mixing. All determinations were carried out in triplicate. The antioxidant activity is reported in *μ*mol of Trolox equivalents per gram matrix (*μ*mol TE/g).

### Ferric reducing antioxidant power (FRAP) assay

This method is based on the reduction, at low pH, of a colorless ferric complex (Fe^3+^- TPTZ) to a blue-colored ferrous complex (Fe^2+^-TPTZ) by the action of electron-donating antioxidants. The ferric reducing power of polyphenol-rich extracts was performed in a 96-well microplate using the FRAP assay (Szeto et al. 2002) with minor modifications. The working FRAP reagent was prepared daily by mixing 10 volumes of 30 mmol/L acetate buffer, pH 3.6, with 1 volume of 10 mmol/L TPTZ in 40 mmol/L hydrochloric acid, and with 1 volume of 20 mmol/L FeCl_3_. A standard curve was prepared using various concentrations of FeSO_4_.7H_2_O. All solutions were used on the day of preparation. FRAP solution (175 *μ*L), freshly prepared and warmed to 37°C, was added to three replicates of each sample (25 *μ*L), while the same volume of acetate buffer was added to the fourth one (blank). The reaction mixture was incubated for 30 min at 37°C, and then the absorbance was measured at 593 nm. The absorbance of the blank was subtracted from the absorbance of the samples. In this assay, the reducing capacity of the samples tested was calculated with reference to the reaction signal given by a Fe^2+^ solution. FRAP values are expressed as mmol Fe^2+^/g of dry matrix.

### Stability of polyphenols bound to protein-rich matrices at 4, 20, and 37°C

Polyphenol stability was measured for a representative subset of the protein-polyphenol matrices; orange- or yellow-fleshed sweet potato flours complexed with blackcurrant (OSP-BC, YSP-BC) and defatted soy flour, light peanut flour, or rice protein concentrate complexed with purple sweet potato extract (DSF-PSP, LPF-PSP, and RPC-PSP). Polyphenols captured in the matrices were gauged over time up to 6 months of storage at 4, 20, or 37°C. Three random samples of each matrix were analyzed, at each of the following time points: 0*,* 2, 4, 8, 12, 16, 20, and 24 weeks of storage at each of the three temperature treatments, for total phenolic content. To minimize any error due to handling during the experiment, three replicates of a sample stored at −20°C were analyzed at each time point representing initial concentration. Results are expressed as percentage of the initial concentration.

### Proximate and descriptive sensory analyses of polyphenol-fortified matrices

One sample from each sweet potato matrix complexed with blackcurrant (OSP-BC, YSP-BC) and one sample of rice protein concentrate complexed with purple sweet potato extract (RPC-PSP), as well as the untreated carriers which had not been complexed with polyphenols (OSP, YSP, and RPC) were packaged in plastic sample bags and shipped frozen to Medallion Laboratories (Minneapolis, MN). Analysis was performed in accordance with AOAC methods for total carbohydrates, ash, moisture, proteins, dietary fiber, fat, and calories per 100-g sample. Methods for these calculations are detailed on the Medallion Labs website (www.medlabs.com).

Similarly, samples of complexed matrices (OSP-BC, OSP-BB, OSP-MG, and RPC-PSP), and untreated OSP and RPC were packaged in plastic sample bags and delivered to Sensory Spectrum Inc. (Kannapolis, NC). Samples were held refrigerated and removed from refrigeration approximately 30 min prior to evaluation. Panelists (five trained persons) were provided approximately one teaspoon of each product in a one oz. cup, with more available in case needed. Panelists evaluated the organoleptic properties of the samples (flavor, taste, and odor) as a dry powder and were allowed to sip water to combine with the powders in the mouth if desired to test the texture.

### Statistics

Statistical analysis was performed with GraphPad Prism v6 (GraphPad Software, Inc., La Jolla, CA). A one-way ANOVA analysis and a Tukey's post hoc multiple comparison test were done with a significance threshold of 0.05.

## Results and Discussion

### Polyphenols captured in fortified matrices

Four sources of polyphenol-rich juices were used in this study; blackcurrant (BC), blueberry (BB), muscadine grape (MG), and purple sweet potato (PSP). Fruit juice concentrates were diluted 1:3 with water due to the high viscosity which otherwise interferes with the sorption process, while purple sweet potato juice was used at full strength without dilution. The total phenolic content (TP), total monomeric anthocyanins (ANC), and % dry matter content in the juices before complexing with protein-rich flours are provided in Table1. TP content ranged from 0.63 ± 0.02 mg/mL (PSP) to 9.08 ± 0.22 mg/mL (BC), measured as gallic acid equivalents. ANC was highest in BC (3.35 ± 0.17 mg/mL) and lowest in PSP (0.11 ± 0.01 mg/mL as cyanidin-3-glucoside equivalents).

**Table 1:** Dry matter, total phenolics, and total monomeric anthocyanin content in the polyphenol-rich sources (mg/mL), and in the complexed polyphenol-fortified matrices (mg/g dry weight)

Sample	Total Phenolics^1^	Anthocyanins^2^
Polyphenol-rich sources		
BC (16.2 ± 0.3% DM)	9.08 ± 0.22	3.35 ± 0.17
BB (16.8 ± 0.1% DM)	3.42 ± 0.03	0.51 ± 0.03
MG (16.6 ± 0.1% DM)	3.24 ± 0.20	0.43 ± 0.03
PSP (2.7 ± 0.9% DM)	0.63 ± 0.02	0.11 ± 0.01
Polyphenol-fortified matrix		
OSP-BC	34.03 ± 7.58^a^	5.40 ± 0.22^b^
OSP-BB	23.59 ± 1.76^b^	2.58 ± 0.09^c^
OSP-MG	14.23 ± 2.85^c^	1.95 ± 0.03^c^
YSP-BC	23.38 ± 3.12^b^	6.54 ± 0.71^a^
YSP-BB	22.88 ± 2.34^b^	2.55 ± 0.24^c^
YSP-MG	10.56 ± 3.48^d^	2.01 ± 0.03^c^
DSF-PSP	9.11 ± 0.38^d^	1.21 ± 0.11^cd^
LPF-PSP	6.09 ± 0.19^d^	0.77 ± 0.14^d^
RPC-PSP	9.46 ± 1.45^d^	1.27 ± 0.04^cd^

1 Total phenolics by Folin–Ciocalteu assay, calculated as gallic acid equivalent.

2 Anthocyanins by pH differential assay, calculated as cyanidin-3-*O*-glucoside equivalent.

DM, dry matter content. Juices: BC, blackcurrant; BB, blueberry; MG, muscadine; PSP, purple sweet potato. Flour matrices: OSP, orange sweet potato; YSP, yellow sweet potato; DSF, defatted soy flour; LPF, light roast peanut flour; RPC, rice protein concentrate. Values are means of 4 determinations × 3 replicates ± standard deviation, Values with different superscript letters within the same column are significantly different (*P *<* *0.05).

**Figure 1:**
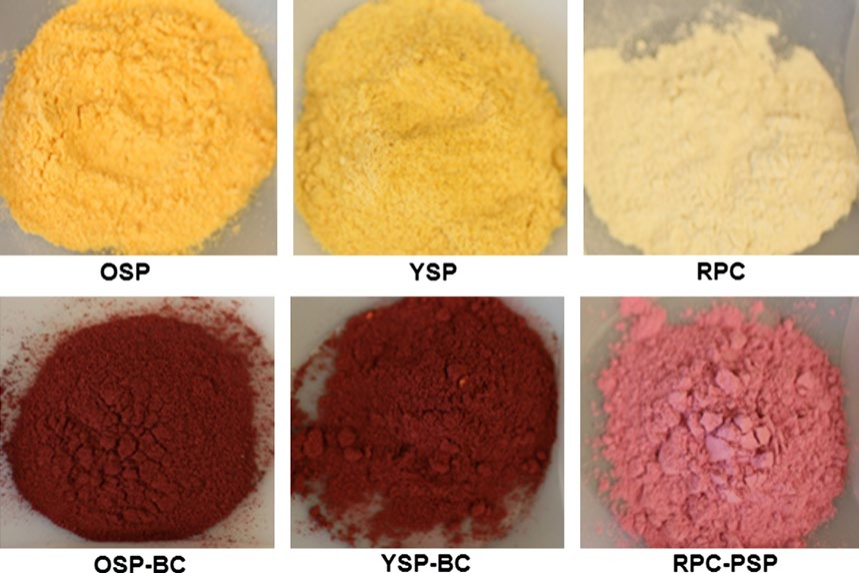
Representative samples of a selection of protein-rich matrices, before (top row) and after (bottom row) polyphenol fortification. OSP, orange sweet potato flour; YSP, yellow sweet potato flour; RPC, rice protein concentrate; BC, black currant; PSP, purple sweet potato.

In these experiments, sweet potatoes were used either as a protein- and fiber-rich flours (OSP or YSP) to capture and stabilize various fruit juice polyphenolics (BC, BB, or MG), or as the source of polyphenols (PSP) to be complexed with other edible proteins (DSF, LPF, RPC). Nine uniformly colored finely powdered polyphenol-fortified matrices were prepared: OSP-BC, OSP-BB, OSP-MG, YSP-BC, YSP-BB, YSP-MG, DSF-PSP, LPF-PSP, and RPC-PSP. Representative samples of protein-rich matrices, before and after polyphenol fortification, are shown in Fig.1.

The concentrations of TP and ANC bound to sweet potato matrices after complexation with the juices are shown in Table1. The efficiency of sorption in different matrices is dependent on several factors including the concentration of protein and fiber in the untreated flour, and the concentration of polyphenols in the juice source (Roopchand et al. 2012a; Grace et al. 2013). Depending primarily on concentration of polyphenol in the juice source, TP content ranged from 6.09 ± 0.19 mg/g (LPF fortified with PSP) to 34.03 ± 7.58 mg/g (OSP fortified with BC). TP from the flour matrices treated only with water (not fortified with fruit juice polyphenols) measured 0.08 mg/g (RPC), 1.22 mg/g (LPF), 1.56 mg/g (DSF), and 2.5 mg/g (OSP and YSP). ANC concentration ranged from 0.77 ± 0.04 mg/g (LPF fortified with PSP) to 6.54 ± 0.71 mg/g (YSP fortified with BC) (Table1). Orange and yellow sweet potato flours captured polyphenols from blueberry juice at comparable efficiencies (about 23 mg/g TP and about 2.5 mg/g ANC). Blueberry fruits of the most commonly cultivated species, *V. corymbosum*, contain on average of 1.8 mg TP per gram of fresh weight; therefore, only 5.9 g of OSP-BB or YSP-BB will provide an average TP (135 mg) equivalent to a 75 g serving of fresh blueberries (Ehlenfeldt and Prior 2001). Protein-rich matrices (DSF, LPF, and RPC) captured TP from PSP at concentration ranges of 6.09 to 9.46 mg/g GAE, with no significant difference.

### Antioxidant capacity in DPPH, ABTS, and FRAP assays

The antioxidant activities of the nine prepared polyphenol-fortified matrices are shown in Table2. Sweet potato flours fortified with blackcurrant or blueberry had the highest radical-scavenging activities in the DPPH and FRAP assays, followed by those fortified with muscadine grape. The ABTS assay did not show significant differences between sweet potato matrices fortified with any of the fruit sources. There were no significant differences in radical-scavenging capacity for any of the alternative protein-rich matrices (defatted soy flour, light roast peanut flour, or rice protein concentrate) fortified with purple sweet potato juice, but each demonstrated significantly lower antioxidant capacity than sweet potato matrices fortified with fruit polyphenols. The antioxidant activities measured using DPPH and ABTS correlated well with the relative total phenolic content of matrices. Similar correlations between oxygen radical absorbance capacity (ORAC) assay results and total phenolic content of various sweet potato genotypes were previously reported (Teow et al. 2007).

**Table 2:** Radical-scavenging capacity (DPPH, ABTS) and ferric reducing antioxidant power (FRAP) of polyphenol-fortified matrices

Polyphenol-fortified matrix	DPPH *μ*mol/L TE/g^1^	ABTS *μ*mol/L TE/g^1^	FRAP mmol/L F^++^/g^1^
OSP-BC	344.66 ± 14.19^a^	833.11 ± 4.32^a^	1.73 ± 0.28^a^
OSP-BB	297.33 ± 4.16^b^	824.65 ± 5.57^a^	1.51 ± 0.02^ab^
OSP-MG	271.33 ± 18.90^bc^	832.21 ± 16.79^a^	1.08 ± 0.09^c^
YSP-BC	196.66 ± 23.69^d^	840.39 ± 1.78^a^	1.36 ± 0.01^b^
YSP-BB	243.33 ± 93.00^b^	828.92 ± 7.39^a^	0.96 ± 0.14^cd^
YSP-MG	102.66 ± 7.02^e^	839.58 ± 6.38^a^	0.94 ± 0.07^cd^
DSF-PSP	33.333 ± 2.74^f^	74.06 ± 8.85^b^	0.28 ± 0.02^e^
LPF-PSP	26.917 ± 1.84^f^	64.78 ± 1.59^b^	0.21 ± 0.01^e^
RPC-PSP	36.66 ± 3.79^f^	82.67 ± 0.67^b^	0.32 ± 0.01^e^

1 Values are means of 4 determinations × 3 replicates ± standard deviation, expressed as *μ*mol/L Trolox Equivalent (TE)/g by DPPH or ABTS assays or mmol/L FeSO_4_ equivalent (F^++^)/g for FRAP assay.

Values with different letters within the same column are significantly different at *P *<* *0.05. OSP-BC, OSP-BB, OSP-MG, orange sweet potato fortified with blackcurrant, blueberry, or muscadine grape, respectively; YSP-BC, YSP-BB, YSP-MG, yellow sweet potato fortified with blackcurrant, blueberry, or muscadine grape, respectively; DSF-PSP, LPF-PSP, RPC-PSP, defatted soy flour, lightly roasted peanut flour, or rice protein concentrate, respectively, fortified purple sweet potato.

### Stability of polyphenols bound to protein-rich matrices

**Figure 2:**
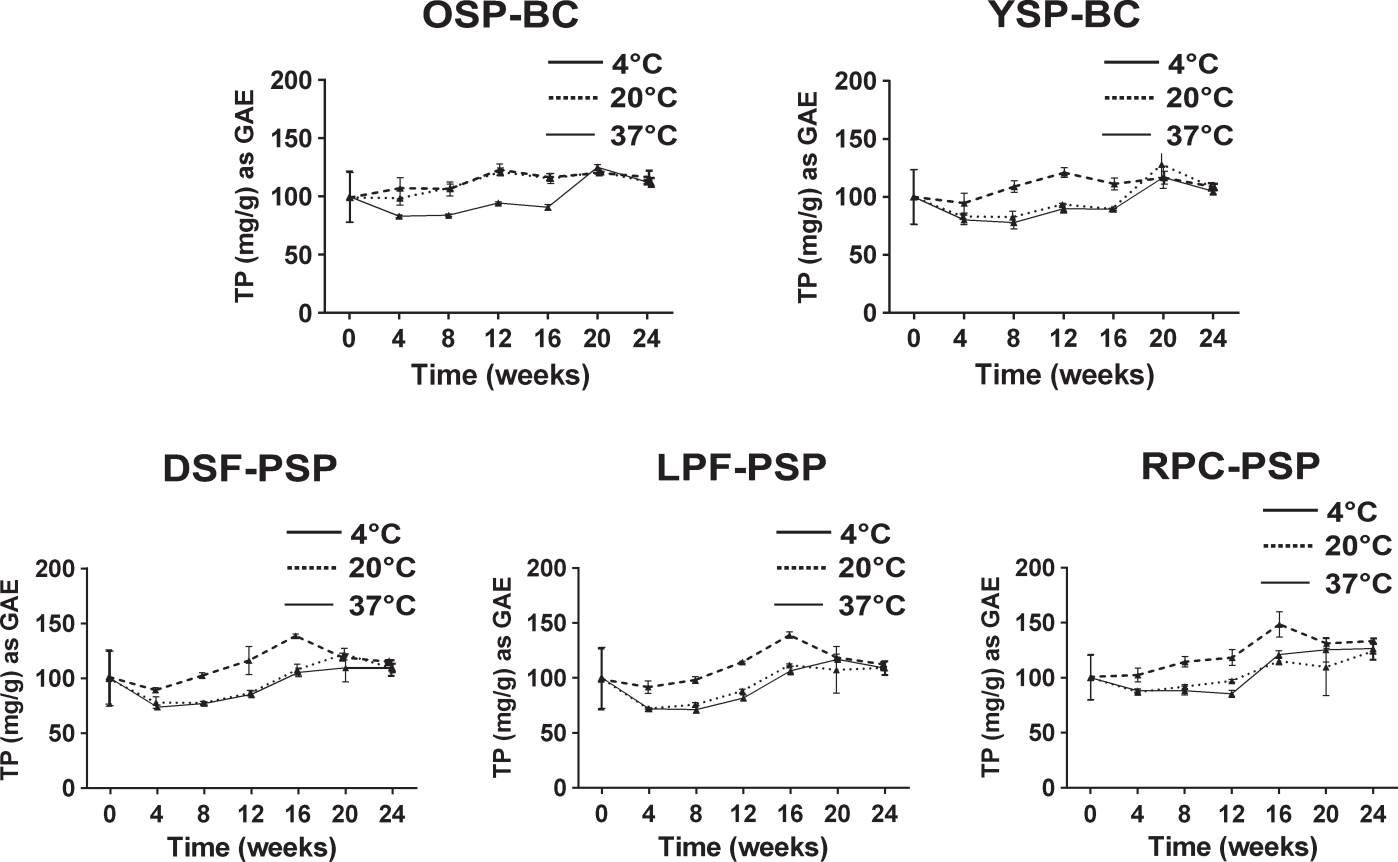
Stability of polyphenols bound to fortified matrices at 4, 20, and 37°C for 24 weeks. There were no significant differences in measured concentrations between times of measurement at *P *<* *0.05. OSP, orange sweet potato flour; YSP, yellow sweet potato four; DSF, defatted soy flour; LPF, light roast peanut flour; RPC, rice protein concentrate; BC, blackcurrant; PSP, purple sweet potato.

Bound polyphenols were extracted from the fortified matrices stored at 4, 20, and 37°C, at time intervals of 0, 2, 4, 8, 12, 16, 20, and 24 weeks, and extracts were quantified for TP. Results were expressed as percentages of initial concentrations (for samples stored at −20°C). In all examined samples, polyphenols were remarkably stable at all tested temperatures over 24 weeks of storage with no significant differences, and with no change in color. There was a slight decrease in TP after 4 weeks of storage at 20 and 37°C, but this decrease was not significant (Fig.2). Stability experiments suggest that the complexation of fruit polyphenols or purple sweet potato polyphenols with protein-rich matrices protected the integrity of polyphenolic components and prevented them from degradation even when incubated at 37°C for 24 weeks. These results are consistent with previous stability tests performed on defatted soy flour or light roast peanut flour complexed with cranberry juice (Roopchand et al. 2012a; Grace et al. 2013), suggesting that the edible protein sources are capable of stabilizing otherwise ephemeral polyphenolic phytochemicals.

### Proximate composition and sensory characteristics of polyphenol-fortified matrices

Proximate chemical analyses were conducted to determine whether the complexation process had any effect the macronutrients in the food matrices. The protein-rich flours were evaluated before and after treatment with polyphenol-rich juices (Table3). Changes in sugar profiles (glucose, fructose, sucrose, and maltose) were noted after complexation, which may indicate that enzymes in the juices hydrolyzed some disaccharides to monosaccharides, or catalyzed the conversion of sucrose into glucose and fructose. An observed overall decrease in total sugars may be a consequence of leaching during the sorption process, as sugars are not typically sorbed to proteins and fibers in the matrix (Roopchand et al. 2012a). The slight increase in total fat and dietary fiber can be explained by the decrease in moisture content of all fortified matrices (Table3).

**Table 3:** Proximate chemical analysis and caloric determination of polyphenol-fortified matrices per 100 g of powder

Assay	OSP	OSP-BB	YSP	YSP-BC	RPC	RPC-PSP
Carbohydrates^1^ (%)	80.44	85.3	79.5	78.8	27.9	27.4
Calories/100 g	366	387	358	385	393	403
Calories from Fat/100 g	11	10	6	21	28	57
Ash (%)	5.28	2.39	6.27	3.23	2.40	4.32
Moisture (%)	4.57	2.36	5.04	3.50	3.32	2.90
Protein (%)	8.64	8.8	8.59	8.99	63.2	69
Total Fat (%)	1.07	1.15	0.63	2.35	3.15	6.37
Dietary Fiber	19.1	29.9	10.6	20.4	3.7	4.7
Sugars %						
Fructose	3.23	8.78	1.95	10.6	<0.1	0.81
Glucose	2.97	7.62	2.72	8.24	0.457	0.96
Sucrose	26.5	5.37	11.8	2.38	<0.1	2.21
Maltose	12.1	2.86	17.4	3.02	8.5	2.22
Lactose	<0.1	<0.1	<0.1	<0.1	<0.1	<0.1
Total sugars (%)	44.8	24.6	33.9	24.2	8.96	6.2
Starch^2^ (%)	19.4	20.8	35.0	37.3	15.2	6.5

OSP, orange sweet potato flour; YSP, yellow sweet potato flour; RPC, rice protein concentrate; OSP-BB, orange sweet potato fortified with blueberry; YSP-BC, yellow sweet potato fortified with blackcurrant; RPC-PSP, rice protein concentrate fortified with purple sweet potato.

1 Carbohydrates were calculated as difference of 100-Protein-Ash-Fat-Moisture.

2 Starch = Carbohydrates – Dietary Fiber – Total Sugars.

Descriptive sensory analysis was performed on the complexed protein-polyphenol matrices, and the untreated flour matrices (without polyphenol complexation) in order to understand the magnitude and type of differences that would be perceived by consumers (Table4). The degree of difference score (DOD) gave qualitative information about distinguishing features and any differences seen. The DOD scale is a 0–10 rating indicating how different a product is from a reference product or control, with 0 meaning no difference and 10 being noticeably different. All tested matrices got a score of 9–10, which indicates extremely different in flavor and/or appearance from their uncomplexed flour base materials. The orange sweet potato-blueberry matrix (OSP-BB) was scored as less sweet than plain orange sweet potato flour, and berry flavors with low to moderate sourness were noted. Sensory Spectrum expert panels suggested that the flavor of this fortified powder ingredient matrix would be well suited for muffins, waffles, or other grain-based products, especially those with bran. The orange sweet potato flour complexed with muscadine grape (OSP-MG) was rated by the panels to have a flavor and aroma of grape. The experts suggested applications where muscadine/white raisin ingredients are suitable. The orange sweet potato flour complexed with blackcurrant (OSP-BC) was rated as reminiscent of jam (with seeds) and berry fruit leather flavor, and supplemental sweeteners were recommended to enhance its palatability. The rice protein concentrate-purple sweet potato (RPC-PSP) was ranked as less sweet than RPC, with a flavor reminiscent of freeze-dried berries. It was suggested that this fortified powder could work well in food applications such as smoothies, cereal bases, and other foods where berry notes are desirable and consumers would likely find this to be a pleasant flavor.

**Table 4:** Descriptive sensory evaluation of fruit polyphenol-fortified sweet potato matrices and rice protein concentrate fortified with purple sweet potato polyphenols

Sample	OSP	OSP-BC	OSP-BB	OSP-MG	RPC	RPC-PSP
DOD^1^	–	10	9	10	–	10
Appearance	Light orange powder	Bright Red-Blue/Claret Wine powder	Purple-brown powder	Purple-brown powder; lighter than OSP-BB	Light Beige, fine powder	Medium Red-purple, fine powder
Flavor (taste and ordor)	Flavor of cooked sweet potato, cooked carrots, general cooked root vegetables/winter squashes.Moderate, noticeable sweetness but not sourNote: aroma is sweet-like cooked squash aroma	Very sour, retains sweetness.Flavor is cooked black currant juice, dark dried fruit, reminiscent of jam (with seeds), and berry fruit leatherNo sweet potato character.	Less sweet than OSPEarthy flavor, not clearly sweet potato (covers sweet part of sweet potato)- more like potato skinsSome berry skin type notes; not like fleshy part of berries → not clearly berry on its ownLow-moderate sourness	No sweet potato character.Retains sweetness; low sournessFlavor is cooked grape and fermented white grape, some cardboard paper flavorAfter taste is muscadine & golden raisinNote: aroma is berry-grape.	Low flavor intensity reminiscent of cooked rice with some toasted rice notes.Includes low caramelized/sweet aroma notes and slight nutty character.Noticeable sweetness	Some sourness, less sweet.Astringent.General red-fruit berry notes reminiscent of freeze-dried berries.No recognizable sweet potato flavor.No distinct RPC flavor, but has slight fermented grain flavor.Note: has very little aroma; does not smell sweet; has slight paper aroma.
Texture	Very sticky when wet, forms a tight ball/mass in mouth; sticks to roof of mouth	Less sticky than OSP	Less sticky than OSP	Less sticky than OSP	Chalky feel in mouth, leaves chalky film on mouth surfaces	

1 DOD = Degree of difference, 0–10 rating with 0 meaning no difference and 10 being extremely different.

OSP, orange sweet potato flour; RPC, rice protein concentrate; BC, blackcurrant; BB, blueberry; PSP, purple sweet potato.

Dietary intake of fruits and vegetables has been positively correlated with reduced risk of many chronic diseases including atherosclerosis, cancer, diabetes, arthritis, and declines associated with aging (Kaur and Kapoor 2001; Scalbert et al. 2005; Cassidy et al. 2013; Shafiee-Kermani et al. 2013). As a result, consumers are increasingly demanding functional foods that conveniently capture and concentrate the phytoactive components from fruits and vegetables (Muller et al. 2013; Potter et al. 2013). The strategy used in this study to create polyphenol and protein-rich matrices was efficient for preserving the integrity and health benefits of polyphenols. In previous work, grape polyphenol-enriched defatted soy flour showed hypoglycemic activity in obese and hyperglycemic C57BL/6 mice, indicating that the matrix effectively preserved the bioactives from grapes (Roopchand et al. 2012b, 2013). In a TNO gastrointestinal model (TIM), blueberry polyphenol-enriched defatted soy flour showed greater anthocyanin bioaccessibility in the ileal efflux samples, compared to blueberry juice. This indicated that the protein-rich matrix protected the polyphenols during transit through the upper digestive tract for subsequent colonic metabolism (Ribnicky et al. 2014). A metabolomics-based investigation showed that 17 days of supplementation with a blueberry and green tea polyphenol-rich soy protein ingredient led to distinct gut-derived phenolic signature and enhanced ketogenesis in long distance runners following a 3-day period of heavy exertion (Nieman et al. 2013). Therefore, it is expected that these polyphenol protein-rich matrices can find applications as ingredients in functional foods or dietary supplements and provide health and performance benefits.

## Conclusions

Several sweet potato polyphenol-fortified matrices that could be used as functional food ingredients were prepared in this study. Orange and yellow sweet potato flours efficiently sorbed, concentrated, and stabilized the antioxidant polyphenols present in fruit juices (blackcurrant, blueberry, and muscadine grape). The matrices combined the benefits of the inherent phytochemicals in sweet potato, and the highly concentrated anthocyanins and other polyphenols from fruits. Using the same strategy, purple sweet potato polyphenols were complexed with protein-rich matrices (defatted soy flour, light roast peanut flour, and rice protein concentrate) to create combinations rich in both protein and stable polyphenols from sweet potato. The concentrations of polyphenols captured in the matrices were significantly higher than in the original juice sources. Sweet potato matrices concentrated total phenolics 3–7 times over their concentration in the original fruit juices, while the other protein-rich matrices (SPI, PNF, or RPC) concentrated purple sweet potato total phenolics 10 times or more. All tested matrices were able to stabilize and preserve polyphenols for a storage period of up to 24 weeks, and the novel matrices retained their vibrant color, even when stored at 37°C. Sensory evaluation of the created matrices suggested good future potential for use as ingredients in various functional food products.
